# Autistic, Aberrant, and Food-Related Behaviors in Adolescents and Young Adults with Prader-Willi Syndrome: The Effects of Age and Genotype

**DOI:** 10.1155/2017/4615451

**Published:** 2017-12-26

**Authors:** Atsushi Ishii, Hiroshi Ihara, Hiroyuki Ogata, Masayuki Sayama, Masao Gito, Nobuyuki Murakami, Tadayuki Ayabe, Yuji Oto, Asami Takahashi, Toshiro Nagai

**Affiliations:** ^1^Department of Psychiatry, Dokkyo Medical University Saitama Medical Center, Saitama, Japan; ^2^National Hospital Organization Hanamaki Hospital, Hanamaki, Iwate, Japan; ^3^Department of Psychiatry, Dokkyo Medical University School of Medicine, Tochigi, Japan; ^4^Ikezawa Hospital, Saitama, Japan; ^5^Department of Pediatrics, Dokkyo Medical University Saitama Medical Center, Saitama, Japan; ^6^Department of Molecular Endocrinology, National Research Institute for Child Health and Development, Tokyo, Japan; ^7^Nakagawanosato Ryoiku Center, Saitama, Japan

## Abstract

The effects of age and genotype were examined, with regard to the severity of aberrant, autistic, and food-related behaviors in Prader-Willi syndrome (PWS), with an emphasis on the contrast between adolescents and young adults. The Aberrant Behavior Checklist Japanese version (ABC-J), the Food Related Problem Questionnaire (FRPQ), and the Pervasive Developmental Disorders Autism Society Japan Rating Scale (PARS) were administered to 65 PWS patients, including 20 adolescents (ages 12 to 17) and 45 young adults (ages 18 to 29). Significant differences (Mann–Whitney *U* tests) were found in ABC-J (*p* = 0.004) and PARS (*p* = 0.021), with lower scores in adolescents than in young adults. While DEL subgroups showed no significant differences between the two age groups in ABC-J (*p* = 0.063) and PARS (*p* = 0.134), mUPD subgroups showed a statistically significant difference in terms of ABC-J (*p* = 0.007). No significant differences were found between adolescents and young adults, in terms of FRPQ (*p* = 0.163). These results suggest that aberrant and autistic behaviors follow a marked worsening trend from around the age of 18. On the other hand, food-related behaviors give no sign of change at this transitory stage. Young adults with mUPD were found to be significantly more severe than adolescents with mUPD, in terms of aberrant behaviors.

## 1. Introduction

Prader-Willi syndrome (PWS) is a neurodevelopmental disorder with an estimated prevalence of around 1 in 25,000 in epidemiological surveys [1–3]. The clinical features of this syndrome are characterized by neonatal hypotonia, intellectual disability, hyperphagia, progressive obesity, and hypogonadism [4, 5]. The physical manifestations of PWS include short stature, small hands and feet, hypopigmentation, and craniofacial anomalies.

As a contiguous gene syndrome, PWS is caused by either the structural loss of material or the absence of gene expression from the paternally inherited copy of chromosome 15q11-13 region [6]. A considerable number of this region are dependent on genomic imprinting, an epigenetic phenomenon whereby the modification of phenotype is subject to the parent-of-origin's contributions to the gene allele [7]. The loss of expression of this region leads to two phenotypically different disorders. Angelman syndrome (AS) is caused by the abnormality on the maternally inherited genes, and PWS by that on the paternally inherited genes. In marked contrast to PWS, AS is characterized by balance and movement disorders, seizures, frequent laughter, and a particular interest in water as well as severe intellectual disability. The phenotypically two distinct syndromes in humans are considered to be the best exemplars of parent-of-origin genomic imprinting, an inheritance process masking the classical Mendelian inheritance.

The absence of expression of the paternally derived genes in this region in PWS occurs mainly by three mechanisms. The first is a paternal deletion (DEL) of 15q11-13 found in 70% of patients. The second is maternal uniparental disomy 15 (mUPD; when both copies of chromosome 15 are maternally inherited) found in 25% [8]. The remaining mechanism is a defect in the imprinting center, the genomic region that controls the activity of imprinted genes (imprinting defect (ID); 1–3%). The cause of IDs includes a microdeletion in the imprinting center and, therefore, can be inherited. The genetic diagnosis of PWS is mainly conducted by fluorescence in situ hybridization (FISH) or the methylation test. In terms of the patients, who show no deletion by FISH although consistent with PWS by methylation test, microsatellite analysis can be performed to discriminate between mUPD and ID.

The clinical significance of PWS for the understanding of gene-environmental interactions lies in the main point. Chromosome 15, responsible for this disorder, has been identified as one of the seven chromosomes enriched in segmental low copy repeats (LCRs) or duplications [9]. According to ample evidence, individuals, with duplications of 15q11-13, show parent-of-origin-specific differences in phenotypes. Maternally derived duplications have an association with autistic spectrum disorders (ASD), whereas paternal duplications of 15q11-13 do not. This suggests the existence of maternally active genes susceptible to ASD in chromosome 15q11-13 [10–12]. Moreover, individuals with PWS have behavioral symptoms reminiscent of ASD, whose prevalence and severity are higher in those with mUPD than in those with DEL subtype [13, 14]. Such an increased risk of ASD in mUPD, in comparison with DEL, suggests that maternally expressed transcripts play a significant role in the pathogenesis of ASD-like symptomatology [15]. In addition to the ASD phenotype, individuals with PWS tend to show a wide variety of maladaptive behaviors. Those include hyperphagia [16], temper tantrums [17], obsessive-compulsive behaviors [18, 19], repetitive and ritualistic behaviors [20], self-injurious behaviors [21, 22], and hyperactive/impulsive behaviors [23]. Behavioral characteristics of this syndrome have been well studied, most of which examined phenotypical differences between DEL and mUPD. It has been reported that the mUPD subtype has a higher risk for psychotic symptomatology [24, 25].

These findings, dealing mainly with Caucasian patients, were supported by a recent study about Japanese PWS patients, implying that phenotypical differences between the two main genotypes in PWS were not subject to ethnicity [26]. Although the mUPD subtype has a higher risk of autistic behavior and psychosis than the DEL subtype [27], this subtype may have a lower risk for other behavioral symptoms, including food-related problems [28], skin picking [29, 30], and obsessive-compulsive behaviors [31].

Until now, however, little was known about the developmental course of maladaptive behaviors from adolescence to adulthood. As far as hyperphagia in childhood was concerned, the developmental trajectories in PWS were well studied, supporting that feeding difficulties and poor weight gain during infancy are replaced by hyperphagia and associated food-related behaviors around the ages of 2 to 3 years. Individuals with PWS often show other distinct behavioral issues, which seem to develop independent of food-related problems. Such non-food-related issues include temper outbursts, obsessive-compulsive behaviors, self-injurious behaviors, and even psychotic symptoms. Compared to food-related behaviors, much less was known how these issues progress over the course of development after adolescence. The extant literature suggests that problem behaviors describe a chronological curve in a nonlinear manner [32–34]. Those are characterized by slow but steady increasing of severity as children get older, young adults show the uppermost level of severity, and older adults with the gradual alleviation of the behaviors. At the same time, the transition along with growth is presumed to depend on behavioral problems, with a few problems that remain unchanged throughout the patients' lives.

In terms of a behavioral transition in PWS, much consideration should be given to differences in genotypes as well as those in behavioral profiles. According to Vogels et al. [27], the escalation trends lie in the autistic and impulsive behaviors in PWS, which are more severe in mUPD than in DEL, and can manifest themselves later in adolescence. In other words, the behavioral difference between mUPD and DEL cases tends to be unrecognizable in childhood. However, less is known about the effect of genotype on behavioral difficulties in terms of the transitory period from adolescence to adulthood.

Based on the current understanding discussed above, this study attempts to focus on the contrast between adolescents and young adults, with regard to aberrant, autistic, and food-related behaviors. Emphasis is placed on the differences between DEL and mUPD as well as those between the two age groups. The reasons for dealing with adolescence is that behavioral characteristics, such as obsessive-compulsive behaviors, autistic propensities, and psychotic symptoms, tend to be inconspicuous in their childhood and to be recognizable in their adolescence. Equally important is the transitional stage from adolescence to adulthood, since, in this period, individuals with PWS are exposed to two socioenvironmental changes. First, after years of schooling, they enroll in vocationally oriented services for mentally handicapped adults around the ages of 17 to 18. Despite intellectual difficulties, they are expected to become more independent and to take a more active role in their workplace. Second, for their medical care, physicians and psychiatrists, partly or entirely, take the place of pediatricians. When, and how, to transition to an adult medical service depends on the individuals. However, for those who have a complicated medical history like PWS, a smooth transition has adverse factors, like the appropriate provision of information including medical history and medications, building a good relationship of trust, the coverage of health insurance, and so on. Such rapid changes of environmental circumstances may have a considerable impact on the behavioral and emotional aspects of individuals with PWS [35].

## 2. Methods

The entire study protocol was reviewed and approved by the Institutional Review Board of Dokkyo Medical University, according to the World Medical Association Declaration of Helsinki. After obtaining informed consent from patients or their parents, the neurocognitive and behavioral assessment of each patient were carried out.

### 2.1. Subjects

This study enrolled 65 Japanese participants with PWS recruited from the Department of Pediatrics, Dokkyo Medical University Koshigaya Hospital. All patients were diagnosed with PWS using fluorescence in situ hybridization or the methylation test. Informed consent for cytogenetic and/or molecular-genetic studies was obtained from all the individuals. The participants consisted of 20 adolescents (ages 12 to 17) and 45 young adults (ages 18 to 29), including 14 adolescents and 35 young adults confirmed having a DEL involving 15q11-13 and 6 adolescents and 10 young adults confirmed having mUPD of chromosome 15 (Table 1).

### 2.2. Methods

#### 2.2.1. The Assessment of Behavior

A comprehensive behavioral assessment was administered to all participants, with regard to aberrant, autistic, and food-related behaviors. The psychologist (H.O.) in charge of collecting data was not informed of the genetic status of each patient. For each participant, H.O. had from 3 to 8 sessions in order to complete behavioral assessment. After administering all measurements in a face-to-face meeting with the parent of the PWS patients, H.O. checked the sheets they filled in for completeness and accuracy. This was for the purpose of obtaining data with quality better than those collected through mailed surveys.

### 2.3. Measures

#### 2.3.1. Intellectual Ability

The same clinical psychologist (H.O.) administered a Japanese version of the Wechsler Intelligence Scale [36–39] for the measurement of IQ. The assessment was conducted in a similar time period, in a quiet and relaxed atmosphere. When patients showed fatigued expressions, a short break was given or the session was deferred until the next hospital visit. The participants completed all the subtests of the WAIS, and a few showed a negative attitude towards the tests.

#### 2.3.2. Aberrant Behaviors

The Aberrant Behavior Checklist Japanese version (ABC-J) [40] was applied for the assessment of the extent of problem behaviors in individuals with PWS. The ABC-J, a 58-item checklist, takes about 10–15 minutes to complete. According to the factor analyses conducted by Aman et al. [41], there are five subscales: (a) irritability and agitation, (b) lethargy and social withdrawal, (c) stereotypic behavior, (d) hyperactivity and noncompliance, and (e) inappropriate speech. It was found that the ABC is one of the useful tools for the measurement of the problem behaviors of individuals with intellectual disability [42] including autism spectrum disorder [43] and is an effective instrument in measuring treatment responses [42, 44].

#### 2.3.3. Food-Related Behaviors

To assess the severity of food-related behaviors, the Food Related Problem Questionnaire (FRPQ) was administered. This is an informant-based questionnaire to assess eating behaviors in people with PWS, consisting of 16 items, with three subscales: preoccupation with food (P), impairment of satiety (S), and other food-related negative behaviors (N). Examples of the questions are the following: “how often does the person compare the size or content of their meal with others?” (P); “after a normal-sized meal, how often does the person say they still feel hungry?” (S); and “if given the opportunity, how often would the person ‘help themselves' to food which they should not have?” (N). As Russell and Oliver presented, the FRPQ has sufficiently robust psychometric properties to appraise the food-related problems in individuals with PWS [45].

#### 2.3.4. Autistic Symptomatology

Autistic symptomatology was assessed using the Pervasive Developmental Disorders Autism Society Japan Rating Scale (PARS) [46, 47]. When assessing adolescents and adults, 33 items for adolescents are applied for the evaluation of current autistic states. The PARS for adolescents is made up of five clinical subscores consisting of interpersonal skills (6 items), communication (7 items), obsession (6 items), problematic behaviors (11 items), and hypersensitivity (3 items).

By means of a numerical coding system, all data was guarded under strict confidentiality and anonymity. The data was analyzed by SPSS 20J for Windows. Mann–Whitney *U* tests were conducted to make three comparisons: the first between adolescents and young adults, the second between adolescents with DEL and young adults with DEL, and the third between adolescents with mUPD and young adults with mUPD.

## 3. Results

The participants' characteristics are presented in Table 1. Both BMI and IQ are higher in young adults than in adolescents, including the mUPD subgroups (BMI and IQ) and the DEL subgroups (BMI).

Table 2 presents the total scores and subscores of ABC-J in the two age groups and the comparison between the two age groups in three pairs: total adolescents and total young adults, adolescents with DEL and young adults with DEL, and adolescents with mUPD and young adults with mUPD. Mann–Whitney *U* tests were applied for statistical comparisons between the two age groups in terms of the severity of behavioral problems. In terms of total patients, statistically significant differences existed in the total score of ABC-J (median = 18.5, 35.0; *p* = 0.004) and four of its five subscores such as “irritability and agitation” (median = 5.0, 13.0; *p* = 0.004), “lethargy and social withdrawal” (median = 3.0, 7.0; *p* = 0.032), “stereotypic behavior” (median = 0, 1.0; *p* = 0.038), and “hyperactivity and noncompliance” (median = 2.0, 8.0; *p* = 0.004), with lower scores in adolescents than in young adults, respectively.

With regard to the DEL subgroups, no statistically significant differences were found between adolescents and young adults in the total score of ABC-J and subscores, except “irritability and agitation” (median = 5.0, 11.0; *p* = 0.038), with lower scores in adolescents than in young adults.

In marked contrast to DEL, the two age groups with mUPD showed statistically significant differences in the total score of ABC-J (median = 16.5, 77.0; *p* = 0.007) and four of its five subscores such as “irritability and agitation” (median = 5.0, 21.0; *p* = 0.031), “lethargy and social withdrawal” (median = 4.5, 19.0; *p* = 0.011), “stereotypic behavior” (median = 0, 7.0; *p* = 0.011), and “hyperactivity and noncompliance” (median = 1.5, 19.0; *p* = 0.003), with lower scores in adolescents than in young adults, respectively.

Presented in Table 3 are the total scores and subscores of FRPQ in the two age groups of PWS patients. No statistically significant difference was found between the two groups in any of the FRPQ scores. Such lack of significant difference was still true for the three pairs: total adolescents and total young adults, adolescents with DEL and young adults with DEL, and adolescents with mUPD and young adults with mUPD.

Table 4 shows PARS total scores and subscores in the two age groups and the comparison between the two age groups in the three pairs. Adolescents scored lower than young adults in terms of the total score of PARS (median = 9.5, 14.0; *p* = 0.021) and two of its subscores, such as “interpersonal skills” (median = 1.0, 2.0; *p* = 0.008) and “obsession” (median = 2.0, 3.0; *p* = 0.037). Examining the DEL subgroups separately, there was no statistically significant difference between adolescents and young adults in the total score of PARS and subscores, except “interpersonal skills” (median = 1.0, 2.0; *p* = 0.022), with lower scores in adolescents than in young adults. Compared with the DEL subgroups, the mUPD subgroups showed a marginally significant difference between the two age groups in terms of the total score of PARS (median = 10, 21; *p* = 0.056), though the difference did not reach the level of statistical significance.

**Figure 1:**
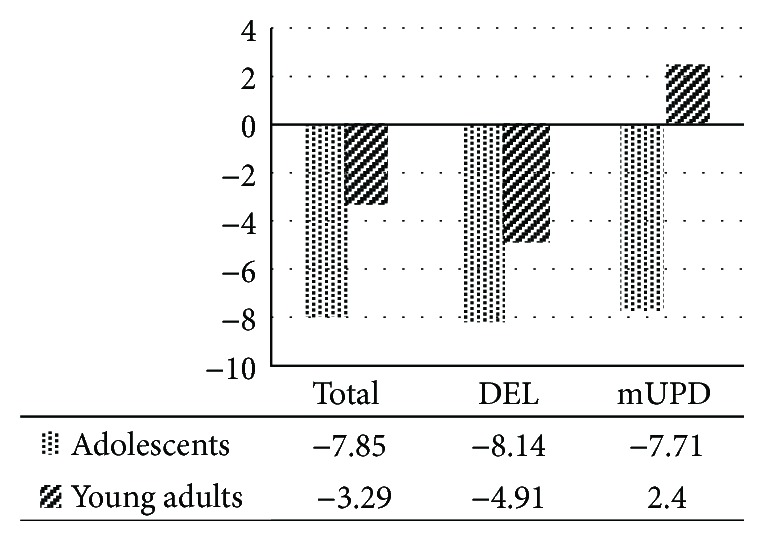
Difference between PARS total score and the cut-off value based on normative date.

Figure 1 presents differences between the PARS total scores of the two age groups in the three pairs and the cut-off value based on normative data cited in [47] collected from 95 individuals. The PARS scores in both total adolescents with PWS and total young adults with PWS were below the cut-off point. These results for the two age groups are true in the same way for the DEL subgroups. On the contrary, the PARS total score for young adults with mUPD was above the cut-off value, in contrast with adolescents with mUPD scoring lower than the cut-off value.

## 4. Discussion

To investigate the severity of problem behaviors, such as aberrant, autistic, and food-related problems, this study attempted to clarify the differences between adolescents and young adults. The results bring out the contrast between food-related problems and non-food-related problems, regarding developmental trajectory. With respect to food-related behaviors, there was no significant difference between adolescents and young adults. On the contrary, non-food-related behaviors were shown to be associated with significant differences between the two age groups. For example, in terms of aberrant behaviors, young adults' behaviors were more severe than adolescents', and such differences between the two age groups were equally true for the mUPD subgroups. Likewise, in terms of autistic behaviors, young adults' behaviors were more severe than adolescents', and such differences between the two age groups can possibly apply to the case of the mUPD subgroups. This is because the PARS total score for young adults with mUPD was above the cut-off value, showing clinically relevant indices of ASD, whereas adolescents with mUPD scored lower than the cut-off value. Though not statistically significant, this data shows a considerable difference between young adults with mUPD and adolescents with mUPD, in terms of the total score of PARS. Compared with mUPD, the DEL subgroups displayed less remarkable differences between adolescents and young adults in aberrant and autistic behaviors, except in one of the five ABC-J subscores (“irritability and agitation”) and in one of the five PARS subscores (“interpersonal skills”).

These findings indicate that food-related behaviors and non-food-related behaviors follow obviously different courses from adolescence to adulthood. The former gives no sign of worsening of symptoms at this transitory stage. On the other hand, aberrant and autistic behaviors follow a marked trend of aggravation from around the age of 18. As for the mUPD subgroups, such a worsening curve is likewise distinct in aberrant behaviors and less distinct, but nonnegligible, in autistic behaviors.

Food-related behaviors are quite unique in the modes of evolution in that they seem to be neither influenced by age nor by genotype. Indeed, several authors [33–35] have already pointed out that a few maladaptive behaviors are lifelong. Perhaps, food-related behaviors are one of those enduring problems. These findings support the results of Pignatti et al. [48] who based on the results of statistical clustering maintained that excessive food intake belongs to a cluster different from another cluster in which some compulsive symptoms and aggressive actions belong.

The results in this study concerning aberrant behaviors are consistent with those of Rice et al. [49], who found no decrease with age of skin picking, frequently observed in PWS, in terms of outward offensive behaviors, such as physical aggression and temper tantrums, people with Prader-Willi syndrome. As for other genetic syndrome groups, such as Down syndrome, Fragile X syndrome, and Williams syndrome, aberrant behaviors decrease under 19 years of age [50, 51]. As for typically developing children, aberrant behaviors such as physical aggression [52] and temper tantrums [53] begin to decline after early childhood. In the present study, maladaptive behaviors in individuals with PWS give signs of worsening, rather than declining, from adolescence to adulthood. Considering these findings about the developmental trajectory of aberrant behaviors, the highest level of severity in PWS is later, in comparison with typically developing individuals and even other types of genetic syndromes.

As for autistic behaviors, the results of this study offer a cautionary suggestion about developmental changes around the age of 18, in regard to PWS in general and mUPD in particular. Around the age of 18, autistic behaviors can manifest symptom exacerbation in individuals with PWS. In contrast to PWS, ASD shows a trend that the severity of autistic symptoms in this disorder is ameliorated during adolescence and the early 20s [54–56]. However, such an improving tendency for the autistic features during adolescents and young adults, found in ASD, cannot necessarily be expected in PWS. On the contrary, a tendency of increased severity should be anticipated in PWS and, above all, mUPD subtypes.

In addition to the differences between the types of problem behaviors, this study also highlights those between the two genotypes, with respect to developmental transition from adolescents to young adults. Compared to young adults with DEL, who showed no significant differences from adolescents with DEL, mUPD young adults were found to be significantly more severe than mUPD adolescents, in terms of aberrant behaviors. Moreover, mUPD young adults scored slightly higher than mUPD adolescents in terms of PARS-based autistic behaviors, though falling short of statistical significance. In this respect, the following possibility should be taken into account that mUPD adults are more prone to autistic and aberrant behaviors than DEL adults. This possibility has already been supported by previous studies. For example, Sinnema et al. [25], based on the examination of 108 adults with PWS at the age of 25 and up, found that problem behaviors in individuals with mUPD were more severe than those in DEL. Examining 100 adults with PWS at the age between 18 and 53, Jauregi et al. [34] also showed that rates of disturbed behavior, such as self-absorbed behavior, communication disturbance, and social-relating difficulties, are higher in patients with mUPD than those with DEL.

According to these findings along with this study, mUPD subtype shows a consistent pattern of increased autism-like symptoms that can be exacerbated later in adulthood, though such a worsening trend is less observable in DEL cases [26]. An exacerbating trend to autistic behaviors in mUPD is more obvious than that in DEL. This supports the prediction that overexpression of maternally imprinted genes in chromosome 15q11-13 is susceptible to ASD. This prediction is also consistent with the currently available evidence that the most frequently identified chromosomal problem in ASD is the copy number variation at this region [13, 57, 58].

Evidently, the current study has a number of methodological limitations. First, the size of the sample is relatively small in that all patients and their parents were recruited from a single institution. Second, this study cannot avoid intergenerational differences, because of cross-sectional comparison between different age brackets. For examining the evolution of phenotypes, behavioral trajectories should be observed, based on longitudinal studies, which trace the same cohort.

## 5. Conclusions

Aberrant and autistic behaviors follow a marked worsening trend from around the age of 18. On the other hand, food-related behaviors give no sign of change at this transitory stage. Young adults with mUPD were found to be significantly more severe than adolescents with mUPD, in terms of aberrant behaviors.

## Figures and Tables

**Table 1:** Participants' characteristics.

	Total		DEL		mUPD		*p* value		
	Adolescents	Young adults	Adolescents	Young adults	Adolescents	Young adults	Total	DEL	mUPD
Number	20	45	14	35	6	10			
Male/female	9/11	26/19	6/8	21/14	3/3	5/5			
Mean age	14.00	22.29	14.29	22.43	13.33	21.5			
Age range	12–17	18–29	12–17	18–29	12–17	319–25			
BMI	22.99	32.58	23.89	32.12	21.02	34.11	0.000^∗∗^	0.012^∗^	0.005^∗∗^
BMI range	14.38–49.45	17.29–72.23	14.38–49.45	17.29–61.81	15.27-28.83	22.31–72.23			
IQ mean (*N*)	44.38 (16)	49.19 (36)	47.2 (10)	49.48 (27)	39.67 (6)	48.33 (9)	0.008^∗∗^	0.271	0.002^∗∗^
IQ range	39–62	39–76	39–62	39–76	39–41	40–62			

*p* values from the Mann–Whitney *U* test. ^∗^*p* < 0.05. ^∗∗^*p* < 0.01.

**Table 2:** ABC-J total scores and subscores in the groups and comparison of the two age groups.

	Total		DEL		mUPD		*p* value		
	Adolescents (*N* = 20)	Young adults (*N* = 45)	Adolescents (*N* = 14)	Young adults (*N* = 35)	Adolescents (*N* = 6)	Young adults (*N* = 10)	Total	DEL	mUPD
	Median	Median	Median	Median	Median	Median			
	(Q1; Q3)	(Q1; Q3)	(Q1; Q3)	(Q1; Q3)	(Q1; Q3)	(Q1; Q3)			
Total score	18.5 (7; 30.75)	35.0 (19.5; 74.5)	18.5 (6.75; 32)	27 (17; 64)	16.5 (6.5; 32.5)	77 (40.5; 91.25)	0.004^∗∗^	0.063	0.007^∗∗^
Subscore									
Irritability and agitation	5 (2.25; 10.5)	13.0 (7.00; 25.50)	5 (2.75; 11)	11 (6; 25)	5 (1.5; 11.5)	21 (9.5; 29)	0.004^∗∗^	0.038^∗^	0.031^∗^
Lethargy and social withdrawal	3 (1; 7.25)	7 (2.00; 16)	2.5 (0.75; 6)	5 (2; 10)	4.5 (9.75)	19 (11; 22.75)	0.032^∗^	0.161	0.011^∗^
Stereotypic behavior	0 (0; 1.75)	1 (0; 5)	0 (0; 2)	0 (0; 3)	0 (0; 1.25)	7 (1; 9)	0.038^∗^	0.314	0.011^∗^
Hyperactivity and noncompliance	2 (1; 7)	8 (3; 17)	3.5 (1; 7.5)	7 (3; 14)	1.5 (0.75; 7.75)	19 (8; 21)	0.004^∗∗^	0.098	0.003^∗∗^
Inappropriate speech	4 (2; 5)	5 (1.5; 7)	3.5 (1.75; 5)	4 (1; 7)	4 (2; 5.25)	6 (4; 7.5)	0.183	0.397	0.147

Q1: 1st quartile; Q3: 3rd quartile. *p* values from the Mann–Whitney *U* test. ^∗^*p* < 0.05. ^∗∗^*p* < 0.01.

**Table 3:** FRPQ total scores and subscores in the groups and comparison of the two age groups.

	Total		DEL		mUPD		*p* value		
	Adolescents (*N* = 20)	Young adults (*N* = 45)	Adolescents (*N* = 14)	Young adults (*N* = 35)	Adolescents (*N* = 6)	Young adults (*N* = 10)	Total	DEL	mUPD
	Median	Median	Median	Median	Median	Median			
	(Q1; Q3)	(Q1; Q3)	(Q1; Q3)	(Q1; Q3)	(Q1; Q3)	(Q1; Q3)			
Total score	34 (23; 47.5)	43 (30.5; 51.0)	37.5 (18.75; 53.5)	44 (35; 51)	33.5 (23; 39.75)	35.5 (19; 52)	0.163	0.278	0.792
Subscore									
Preoccupation with food	11 (7; 14)	11 (6.5; 13)	11.5 (6; 15)	11 (8; 13)	10.5 (8.5; 11.75)	7.5 (2; 13.25)	0.563	0.681	0.313
Impairment of satiety	15.5 (13; 21.75)	19 (16; 21)	14.5 (11.25; 24)	20 (16; 21)	17 (13.75; 18.25)	18.5 (9.75; 23.25)	0.210	0.498	0.492
Composite negative behavior	7 (3; 15)	13 (7.5; 16.5)	9 (3; 16)	14 (9; 19)	6 (2.25; 9.25)	9 (3.5; 15.25)	0.050	0.115	0.428

Q1: 1st quartile; Q3: 3rd quartile. *p* values from the Mann–Whitney *U* test.

**Table 4:** PARS total scores and subscores in the groups and comparison of the two age groups.

	Total		DEL		mUPD		*p* value		
	Adolescents (*N* = 20)	Young adults (*N* = 45)	Adolescents (*N* = 14)	Young adults (*N* = 35)	Adolescents (*N* = 6)	Young Adults (*N* = 10)	Total	DEL	mUPD
	Median	Median	Median	Median	Median	Median			
	(Q1; Q3)	(Q1; Q3)	(Q1; Q3)	(Q1; Q3)	(Q1; Q3)	(Q1; Q3)			
Total score	9.5 (8; 15.75)	14 (10; 21)	8.5 (8; 16.5)	13 (9; 18)	10 (7.75; 17.5)	21 (18.5; 27.5)	0.021^∗^	0.134	0.056
Subscore									
Interpersonal skills	1 (0; 2)	2 (1; 4)	1 (0; 2)	2 (1; 3)	2 (0; 2.5)	4 (2; 7)	0.008^∗∗^	0.022^∗^	0.056
Communication	4 (3; 5.75)	4 (3.5; 6)	4 (3.75; 6.25)	4 (3; 5)	3 (2.75; 5.75)	5 (4; 7.25)	0.535	0.701	0.093
Obsession	2 (1; 3)	3 (2; 4)	2 (1; 3)	2 (1; 4)	1.5 (0.75; 4)	4 (2; 5)	0.037^∗^	0.169	0.093
Problematic behaviors	3 (1; 5)	5 (2; 8)	3 (1; 5.25)	4 (2; 7)	3.5 (0.75; 6.5)	8 (5.75; 9)	0.115	0.397	0.093
Hypersensitivity	1 (0; 1.75)	1 (0; 2)	0.5 (0; 1.25)	1 (0; 1)	1.5 (0; 2.25)	1.5 (0; 2.25)	0.551	0.346	0.875

Q1: 1st quartile; Q3: 3rd quartile. *p* values from the Mann–Whitney *U* test. ^∗^*p* < 0.05. ^∗∗^*p* < 0.01.
